# Maintaining face-to-face contact during the COVID-19 pandemic: a longitudinal qualitative investigation in UK primary care

**DOI:** 10.3399/BJGPO.2021.0036

**Published:** 2021-09-01

**Authors:** Andrew Turner, Anne Scott, Jeremy Horwood, Chris Salisbury, Rachel Denholm, Lauren Scott, Geeta Iyer, John Macleod, Mairead Murphy

**Affiliations:** 1 National Institute for Health Research Applied Research Collaboration West (NIHR ARC West), University Hospitals Bristol and Weston NHS Foundation Trust, Bristol, UK; 2 Population Health Sciences, University of Bristol, Bristol, UK; 3 Centre for Academic Primary Care (CAPC), University of Bristol, Bristol Medical School, Bristol, UK; 4 NIHR Bristol Biomedical Research Centre (BRC), Bristol, UK; 5 Bristol, North Somerset, and South Gloucestershire Clinical Commissioning Group (BNSSG CCG), Bristol, UK

**Keywords:** COVID-19, face-to-face consulting, general practitioners, referral and consultations, primary healthcare, general practice

## Abstract

**Background:**

In March 2020, the COVID-19 pandemic required a rapid reconfiguration of UK general practice to minimise face-to-face contact with patients to reduce infection risk. However, some face-to-face contact remained necessary and practices needed to ensure such contact could continue safely.

**Aim:**

To examine how practices determined when face-to-face contact was necessary and how face-to-face consultations were reconfigured to reduce COVID-19 infection risk.

**Design & setting:**

Qualitative interview study in general practices in Bristol, North Somerset, and South Gloucestershire.

**Method:**

Longitudinal semi-structured interviews with clinical and managerial practice staff were undertaken at four timepoints between May and July 2020.

**Results:**

Practices worked flexibly within general national guidance to determine when face-to-face contact with patients was necessary, influenced by knowledge of the patient, experience, and practice resilience. For example, practices prioritised patients according to clinical need using face-to-face contact to resolve clinician uncertainty or provide adequate reassurance to patients. To make face-to-face contact as safe as possible and keep patients separated, practices introduced a heterogeneous range of measures that exploited features of their indoor and outdoor spaces, and altered their appointment processes. As national restrictions eased in June and July, the number and proportion of patients seen face to face generally increased. However, the reconfiguration of buildings and processes reduced the available capacity and put increased pressure on practices.

**Conclusion:**

Practices responded rapidly and creatively to the initial lockdown restrictions. The variety of ways practices organised face-to-face contact to minimise infection highlights the need for flexibility in guidance.

## How this fits in

During the first wave of COVID-19 in the UK there was wide regional variation in infection rates; the South West had one of the lowest infection rates in the country. GP practices in Bristol, North Somerset, and South Gloucestershire implemented measures to allow COVID-19 patients, low-risk and shielded patients to be seen by their normal doctor, while making face-to-face contact with patients as safe as possible. The very different ways that practices organised face-to-face contact to minimise infection highlights learning for the future for low-prevalence pandemic regions who wish to keep all services in-house on order to maintain continuity of care, rather than directing patients with symptoms consistent with COVID-19 (for example, cough or fever) to specific locations ('hot hubs') for treatment.

## Introduction

In March 2020, the COVID-19 pandemic^1^ required a rapid reconfiguration of UK general practice to minimise face-to-face contact with patients to reduce infection risk.^2,3^ Practices were advised by NHS England to move to a ‘total triage’^4^ access model, using a combination of telephone, online, and video consultations,^5–7^ and clinically extremely vulnerable patients were advised to ‘shield’, avoiding all but essential contact.^8^ However, some face-to-face contact remained necessary.^9^ NHS England later reminded GPs of the need to offer face-to-face consultations, which caused some controversy^10,11^ since it implied that GPs were putting patients at risk through avoiding face-to-face contact in situations where it was both feasible and clinically appropriate. GPs argued strongly that this was not the case.^12,13^

Royal College of General Practitioners (RCGP) and the British Medical Association (BMA) guidance on workload prioritisation published in April 2020^9^ adopted a red–amber–green (RAG) rating, in which green activities must continue whatever the prevalence of COVID-19, amber activities should continue if practices had the capacity, and red activities could be deferred if there was high prevalence. Green activities included long-term condition reviews for patients at high risk (for example, poorly controlled diabetes), whereas red activities included, for example, spirometry, ear syringing, and non-urgent screening such as NHS Health Checks.

Working within this national guidance, practices determined locally what they could safely defer, move to remote care, or continue face to face. COVID-19 infection rates varied widely throughout the UK and some high-prevalence regions implemented COVID-19 hot hubs, where suspected COVID-19 patients were directed to specific locations for treatment.^14,15^ However, Bristol, North Somerset, and South Gloucestershire Clinical Commissioning Group (BNSSG CCG) had relatively low prevalence. In May 2020, prevalence in the South West was estimated to be the lowest in England, at 0.06%, compared with the highest in London at 0.20%.^16^ Practices implemented a number of measures to allow patients with suspected COVID-19, as well as both low-risk and shielding patients, to continue to be seen in their local GP practice.

COVID-19 is likely to change the way general practice works in the future. The changes to how consultations take place call for reflection on what innovations to retain post-pandemic. This article examines how, during the first wave of the COVID-19 pandemic, practices in a low-prevalence region determined when face-to-face contact was necessary and how face-to-face contact was made possible.

## Method

Longitudinal semi-structured individual interviews with clinical and managerial staff from general practices in BNSSG CCG were carried out. These were collected as part of the mixed-methods RAPCI Study (Rapid COVID-19 intelligence to improve primary care response), the results of which are reported elsewhere.^17^

### Recruitment

Practices in BNSSG were provided with study information by the CCG. Expressions of interest were received from practices and a selection were recruited, ensuring a mix of practice sizes and deprivation scores. Practice staff were approached to take part through the practice manager or research lead.

### Data collection

Longitudinal interviews were conducted with practice staff in four rounds (approximately 2–3 weeks between interviews) between May and July 2020, by three authors. The same staff were not necessarily interviewed in each round (see Table 1). Interviews used a flexible topic guide (see Supplementary File 1) to investigate how practices dealt with implementing new consultation models, challenges faced, and solutions developed. This was refined iteratively as interviews and preliminary analysis progressed. Verbal, recorded consent was obtained from all participants. Data collected were held securely at the University of Bristol. Interviews were audio-recorded and lasted 10–40 minutes. Audio-recordings were transcribed via approved services, and data anonymised.

**Table 1.** GP practice and participant characteristics

**GP practice**	**Patient list size^a^**	**IMD** **quintile^b^**	**Interview participants**
**Round 1** **13–27 May**	**Round 2** **28 May–13 Jun**	**Round 3** **15 Jun–2 Jul**	**Round 4** **3 Jul–27 Jul**
1	Medium–large	1	GP1, PM1	GP1	GP1	NM1
2	Small–medium	2	GP2, PM2	GP2	GP2	GP2
3	Medium	3	GP3, PM3	NM9	GP3	NM9
4	Medium–large	5	GP4	GP4	GP4	NM2
5	Small	1	GP5, PM4	0	GP5	NM3
6	Very large	5	GP6, PM5	GP6	GP6	GP6
7	Medium	5	GP7	GP7	GP7	GP7
8	Small–medium	5	GP8, PM6	GP8	NM4	GP8
9	Very large	5	GP9	GP9	GP9	NM5
10	Small–medium	5	GP10, PM7	GP10	GP10	GP10
11	Small	1	GP11	GP11	GP11	GP11
12	Very large	3	GP12	GP12	GP12	0
13	Small	5	GP13	GP13	GP13	GP13
14	Medium	5	GP14, PM8	GP14	GP14	NM6
15	Small	5	0	GP15	GP15	GP15
16	Small	3	0	GP16, PM9	0	GP16, PM9, NM7
17	Small–medium	3	0	GP17, PM10	GP17	GP17
18	Small	1	0	GP18, PM11	GP18	GP18
19	Small–medium	2	0	GP19	GP19	GP19
20	Medium	2	0	GP20	GP20	GP20
21	Small	1	0	GP21	GP21	NM8
**Total interviews**			**22**	**23**	**20**	**22**

a Small:<10 000 patients; small–medium: 10 000–15 000 patients; medium: 15 000–20 000 patients; medium–large: 20 000–25 000 patients; large: 25 000–30 000 patients; very large: >30 000 patients. ^b^Index of Multiple Deprivation (IMD) quintile 1 = most deprived and 5 = most affluent.

GP = general practitioner. IMD = Index of Multiple Deprivation. PM = practice manager. NM = nurse manager, advanced nurse practitioner, or senior nurse.

### Analysis

Interviews were fully transcribed and anonymised, then coded using QSR International NVivo (version 12) software. Thematic analysis^18^ was used to examine staff descriptions of when and how face-to-face contact with patients was maintained. Four authors established an initial coding framework and three authors double-coded six interviews (two each) to ensure a coding consensus and to maximise rigour. All data relating to maintain face-to-face contact were then coded into the final coding framework.

## Results

### Practice and participant characteristics

Eighty-seven interviews were conducted with 21 GPs, 11 practice managers, and nine nurse managers from across 21 practices, which covers 25% of the CCG’s practices. Practice and participant characteristics are shown in Table 1.

Two key themes were developed from the analysis: ‘When face-to-face contact was necessary’; and ‘How face-to-face contact was made possible’. Subthemes outline the key elements of these themes. Findings are illustrated using anonymised verbatim quotes.

### When face-to-face contact was necessary

Face-to-face contact remained necessary for multiple reasons. First, there were non-clinical reasons to see some patients face to face, such as when patients lacked access to technology or were not able to communicate effectively remotely:

*'* *I know that is a bit of a risk* [bringing patients into the practice]*, but we deem it an acceptable risk, and the patient will often deem it an acceptable risk. So, we probably have a lower threshold to bring in those who struggle with English as a first language, those who are more elderly* [older]*, and maybe have a range of symptoms, which is hard for them to articulate.* *'* (GP, practice-18)

Beyond this, the circumstances where face-to-face contact was necessary included both routine and acute care.

#### Routine care

All practices maintained contact with patients with long-term conditions, prioritising face-to-face contact with those who needed examination or treatment, and dealing with others remotely. Typically, this meant focusing face-to-face contact on patients with poorly controlled conditions, such as those *'* *not responding to a change in medication or* [who] *we thought they were sicker* *'* (NM, practice-14), as well as patients with multiple conditions and patients *'* *most at risk of both complications from the*[ir] *condition but also complications of COVID*[-19] *if they get it* *'* (GP, practice-12). To prioritise patients, some practices sent questionnaires (by short message service [SMS] text message, post, and email) to assess the needs of their patients with long-term conditions and identify those needing face-to-face assessment.

Other routine face-to-face activities also continued for those most in need, in line with BMA and RCGP guidance. The ability of practices to continue routine work varied according to their circumstances. For example, all smear tests and long-acting reversible contraception (LARC) services were cancelled at one practice because they initially had problems obtaining personal protective equipment (PPE), whereas another practice decided to continue LARC services (despite it being advised to pause them) because they had patients who were *'* *vulnerable sex workers* *'* (PM, practice-18).

#### Acute care

Staff commonly noted that face-to-face contact was required for patients with urgent or acute problems that required a physical examination, such as abdominal pain, or where GPs needed more holistic non-verbal information about a patient. Face-to-face contact was also thought necessary when there was a suspicion that a patient may need admitting to hospital, but it was not possible to *'* *make that judgement over a phone call* *'* (GP, practice-12). Equally, face-to-face contact often became necessary to resolve GP uncertainty following one or more remote consultations:

*'*[A colleague] *was saying,* *"* *I’ve kicked the can long enough down the road with these people. I’ve tried the stuff that I wanna go through with them but now I do need to press the flesh and satisfy myself that I’ve done everything.* *"* […] *"* *I’ve tried the telephone, I’ve tried the video, and what I thought might work hasn’t worked, so let’s get* [the patient] *in and go into it a little bit deeper.* *"'* (PM, practice-6)

Here a GP is reported as wanting to see the patient because face-to-face contact was needed to provide more clinical or diagnostic information. Other GPs decided face-to-face contact became necessary because patients *'*[were] *not feeling reassured enough by the examination over a video link or the telephone call* *'* (GP, practice-12).

#### Decisionmaking process for booking a face-to-face appointment

Views about when face-to-face contact was necessary were reasonably consistent (as above); however, GPs found it challenging to establish when the benefits of face-to-face contact outweighed the risks:

*'* *I am weighing up the trade-offs of whether she* [the patient] *has anything to gain by me seeing her or not* […] *it’s emotionally quite difficult to be in a situation where you seeing someone face-to-face may actually be harmful for them.* […] *"* *I want to help you and me seeing you may not help you!* […] *It may actually harm you*."' (GP, practice-15)

In some practices, the process of double-checking with colleagues was introduced to help clinicians make these decisions consistently:

*'* *If any of us want to see a patient face* *to* *face, we send a quick pop* *-* *up around to our colleagues that are working that day, not so much to ask permission but just saying,* *"* *D* *o you think it’s reasonable? Can you think of any other way we can manage this?* *"* *Just so we’re not getting one doctor who is doing things differently from everyone else.* *'* (GP, practice-9)

GPs also recognised that minimising face-to-face contact to the degree achieved in March to May 2020 was an *'* *extemporising* *'* (GP, practice-19) and temporary measure. As infection rates reduced from June to July, GPs increased face-to-face contact *'* *particularly in cases where the clinical risk is maybe moderate to high, rather than it was just high before* *'* (GP, practice-2). GPs also reported relying more on their own judgement for when face-to-face contact was necessary rather than double-checking with colleagues:

*'* *We’ve relaxed our rules a little bit* […] *we had this protocol where we would check with anyone else before bringing someone in, and we’re not doing that anymore, we’re bringing them in ourselves.* *'* (GP, practice-9)

In part, this was owing to infection control procedures becoming embedded leading staff to *'* *feel that we know what we’re doing* *'* (GP, practice-7). Equally, GPs were aware that problems may be being missed and the balance between infection risks and risks of remote management were shifting, making it increasingly important to re-establish face-to-face contact. As one GP described, *'* *we can’t wait on this anymore, let’s* [...] *have a look at them face to face* *'* (GP, practice-9).

### How face-to-face contact was made possible

To make face-to-face contact as safe as possible for staff and patients, practices adapted their buildings and processes to increase infection control measures, particularly for shielding patients.

#### Zones

Different ways of ‘zoning’ practices were used to separate patients with or without suspected COVID-19. For practices with multiple sites, this separation was sometimes achieved by establishing dedicated ‘hot’ sites for suspected COVID-19 patients and ‘cold’ sites for patients without COVID-19 symptoms:

*'...* *our hot site* [is] *where we will only see patients with respiratory symptoms.* […] *Therefore, that has made all our other sites what we call cold sites, where we are not seeing respiratory symptom patients. So then we minimise that risk for patients who have got non-respiratory type symptoms.*' (GP, practice-6)

Hot and cold sites could also allow routine care to continue by dedicating some sites to specific tasks, such as blood tests for shielding patients:

*'*[shielding patients] *go to one of our sites, which is specially designated as a clean site* […] *they’re just doing shielded bloods.* *'* (GP, practice-9)

Single-site practices often introduced hot-and-cold zoning within buildings, exploiting features like multiple floors or multiple entrances to control patient movement within the practice building. Not all practices had these options however, and instead (or as well) practices ‘zoned’ their hours to keep patients (in particular, those shielding) temporally separate:

*'...* *our building hasn’t got another door!* […] *what we have done is bring our shielded patients in by appointment, first thing in the morning, so the building has just been cleaned. They are separated out by quite a lot of time* *.'* (GP, practice-13)

#### Building modifications

Many practices made significant modifications to their buildings. For instance, one practice had *'* *clinical flooring installed …* [to create] *our* *"* *dirty* *"* *zone*' (GP, practice-4), another had *'* *a new tarmac path* [...] *around the side of the building* […] *to provide safe access for patients* *'* and new stud walls to divide a large treatment room in two. Many others installed perspex screens at reception. Furthermore, to facilitate social distancing, practices took measures such as *'* *strip*[ping] *consulting rooms bare* *'* (GP, practice-4), reorganising desks in reception areas, and having minimal chairs for patients in waiting areas.

#### Outside spaces

Outside spaces were used to keep patients separate. Many practices used their car parks as a waiting area, either by *'* *calling* [waiting patients ...] *straight in from the car* *'* (GP, practice-9), or adding seating outside. Practices also used their car parks as treatment areas by erecting gazebos and creating private areas within these for simple assessments and taking blood. These measures were used for both acute and routine clinical activities, including for shielding patients, and created, for staff, a safer alternative to home visits.

*'...* *it’s just the additional risk COVID-wise going into someone’s house, so we’re happier to do it in the car. So if we can get people to come to the car park, coming to the sectioned off bit, so there’s patient privacy, and then doing their bloods in the car.* *'* (GP, practice-17)

#### Home visits

Home visits were used as another way to maintain necessary face-to-face contact, especially with vulnerable or shielding patients. Although home visits were noted as being more time-consuming, visits to shielding patients were used when it was difficult to minimise the infection risks of coming to the practice (for example, where it was not possible to repurpose car parks, or where practice buildings lacked multiple entrances), or when particular patients were anxious about leaving their house, or unable to arrange transport. Many practices also had nurses doing home visits for shielding patients, even in practices with little history of nurse visits. These visits were used, for example, to change dressings or take blood.

#### Rationalising appointment processes

The reconfiguration of practices was accompanied by new appointment processes to further minimise the number of people in the building at one time and to allow for extra cleaning and changing time (into and out of PPE) between appointments:

*'* *We’ve spaced out the appointments* […] *spread throughout the day so we haven’t got too many people in the waiting room. And we’ve adjusted our rotas so we’ve got no more than two nurses in at any one time.* *'* *(*PM, practice-16)

As demand for routine appointments increased and became more challenging to manage, some practices established clinics for seeing certain types of patients in dedicated blocks:

*'...* *we’ve got less appointments on the system ‘cause the appointments are longer. So, I think what we’re gonna do is we’re gonna actually have a HCA* [healthcare assistant] *doing a blood clinic every day, so that simple bloods can just go into that* […] *otherwise we’re not gonna be able to churn through the number of just bloods that we need to do* *.'* (GP, practice-7)

A further innovation to improve the efficiency of face-to-face contact was to combine as many tasks as possible when patients visited, creating a *'* *one-stop shop* *'* (GP, practice-1). For example, taking blood from patients immediately following a GP consultation; combining multiple long-term condition reviews that a patient may need; and doing the face-to-face components of overdue or upcoming long-term condition reviews if a patient visited for a routine blood test

*'...* *when someone comes in for a blood test, the nurses are now looking up at the pop-ups to see what other things are due.* […] *Basically, what we’re trying to do is everything that that patient may need.* […] *We’re just trying to minimise people coming down and if you’re coming down, why not have everything done that you need to have done* *?'* (GP, practice-7)

Practices also adopted strategies to reduce unnecessary face-to-face time during visits. This was often achieved by separating long-term condition reviews into the physical exams, which were done face to face, and the subsequent discussions that could be done remotely:

*'*[patients with diabetes] *come in for their blood test. They’re weighed and measured.* […] *We try and do everything as a “oner” and then, unless there’s some pressing reason, usually they go home then. Then the nurses can speak to them a couple of days later to go through the results and hopefully try and get control of their HbA1c or blood pressure, or whatever that might be.* *'* (GP, practice-18)

#### Challenges as demand increased

The time taken to follow new infection control procedures reduced practices' capacity to meet increasing demand. As the first wave of the pandemic passed and the number of COVID-19 cases in the South West remained low, COVID-19 zones within practices needed to be reclaimed for the care of non-COVID-19 patients:

*'...* *we need our* *"* *hot room* *"* *back, we can’t function without that room as things get busier. So, we’ve had to make a* *"* *hot room* *"* *with a temporary structure in the garden.* […] *we simply haven’t got the space to not do that and increase up to our normal functioning.* *'* (GP, practice-13)

The need to divert clinician time to new tasks, such as additional home visits (noted as being particularly time-consuming owing to infection control measures) or allocating a doctor to suspected COVID-19 patients, was a further barrier to meeting increasing demand for non-COVID care (while also adjusting to total triage access models):

*'...* *we have had to take someone out to do the COVID work. That GP may only see three or four patients in the course of the day. We have also taken out a GP to do all our home visiting* […] *having taken those clinicians away from perhaps being able to churn through the bulk of our clinical work has also led to a bit of pressure as well.* *'* (GP, practice-12)

Moreover, many existing tasks took longer because of the new processes in place. For example, lengthened appointment times, particularly for nurse appointments, meant an overall reduction in the number available.

Measures adopted to make face-to-face contact possible, which staff described in the interviews, are summarised in Table 2.

**Table 2.** Measures taken by practices to make face-to-face contact possible

**Measures taken by practices^a^**	**Examples**
**Zones**	
Physical zoning	Areas of buildings (‘red zones’) used only for suspected COVID-19 patients.Areas of buildings (‘green zones’) used only for patients not suspected of having COVID-19.
Temporal zoning	Shielded patients seen first thing in the morning, before any other patients enter the building.
**Building modifications**	
One-way systems	Routes within practices that avoid staff and patients meeting in corridors where distancing cannot be maintained.
Use of multiple entrances and exits	Side entrances used for access to ‘red zone’, minimising interaction between suspected COVID-19 patients and other patients, and reducing the amount of space necessary to create red zones.
Repurposed spaces	Stripping bare clinic rooms for easier cleaning and semi-permanent changes to buildings (new flooring to expand clinical spaces, new walls to divide larger clinical rooms).
**Home visits**	
Home visits	Shielded or vulnerable patients visited by GPs and nurses, for acute and routine care.
**Outside spaces**	
Use of practice car parks or gardens	Sections of car parks used as waiting or treatment areas.
**Rationalising appointment processes**	
Longer appointments	Adding 5 minutes before and after treatment-room appointments to allow staff to change into PPE beforehand and clean room afterwards.
Consolidation of face-to-face tasks	Opportunistically doing patients’ blood tests or reviews of long-term conditions if they visit the practice for other reasons.
Segmentation of reviews of long-term conditions	Conducting only essential physical exams or observations face to face, then completing review remotely.

^a^This table is derived from the interview data and summarises the various measures that were described by staff. PPE = personal protective equipment.

## Discussion

### Summary

At the start of the UK’s first lockdown in March 2020, in response to the COVID-19 pandemic, general practice reduced and reconfigured the services available to minimise infection risk to staff and patients. This study presents accounts of the changes that many practices made to maintain necessary face-to-face contact.

Practices worked within national guidance to determine when face-to-face contact with patients was necessary. This included prioritising patients according to clinical need and using face-to-face contact to resolve clinician uncertainty or provide reassurance to patients. To make face-to-face contact as safe as possible and keep patients separated, practices introduced a heterogeneous range of measures that exploited features of their indoor and outdoor spaces, and altered their appointment processes. As the UK passed the peak of the first wave of the pandemic over June and July 2020, services were scaled-up again and confidence seeing patients face-to-face grew. However, the need to maintain processes to enforce social distancing and infection control reduced the available capacity and put increased pressure on practices.

### Strengths and limitations

This is one of the first articles describing how face-to-face contact was maintained during the COVID-19 pandemic. A strength of the study was the ability to capture the dynamic general practice response to the pandemic, using longitudinal interviews, during a time of rapid change. The sample included a mix of practices in relation to patient list sizes and located in areas of different deprivation levels. Conducting 87 longitudinal interviews in 21 practices captured a range of views and allowed for in-depth exploration of the considerable variety of measures put in place and changes over time, with sufficient ‘information power’^19^ being achieved for the core themes presented. Because patient-facing research was paused when this study was started, patients were not interviewed. Furthermore, during the study, the community COVID-19 prevalence in the research area was relatively low and the findings should be interpreted with these limitations in mind.

### Comparison with existing literature

Existing literature on the GP response to delivering services during the COVID-19 pandemic has largely focused on the impact of shifting to mostly remote consultations;^5,7,17,20,21^ for example, data from practices using the *a* *skmyGP* platform (a platform for online triage and consultations) showed a large drop in patient requests for face-to-face contact.^22^ Guidance was rapidly issued on when certain types of consultation may be most appropriate^6^ and subsequently there has been reflection on the innovations that should be retained post-pandemic.^20^ This includes evidence of emerging narratives in the media that are more critical of the trade-offs entailed by a permanent shift to remote consultations as the default.^23^

The findings corroborate those of Sharma *et al*, who conducted an online survey of GPs and practice managers in the East Midlands during May and June 2020 to explore how practices were coping with the pandemic.^12^ They found practices implementing a similar range of measures, to both buildings and processes, to make face-to-face contact possible. This is despite much higher infection rates in the East Midlands (Leicester was the first city in England to be put under new restrictions after a second spike in local COVID-19 infections in June 2020),^24^ demonstrating the likely utility of these measures even in high-infection areas.

The use of these measures was also critically dependent on the time of year. Practices implemented the measures from April–July 2020 over a warm spring and summer. There was a low rate of other viral infections,^25^ whereas in colder months there are more patients with viral infections who may need to be treated as potential COVID-19 patients.^26^ Using outside spaces is less feasible in poor weather.^27^ Instead, practices may require more 'one-stop shop' rationalisation of patient visits to health centres and greater reliance on strategies to separate patients within buildings, further straining practice capacity. The delivery of a successful — and socially distanced — flu vaccination programme in autumn 2020 and the rollout of COVID-19 vaccinations in 2021 required some similar measures to make them possible, such as the use of outside spaces or *'*[configuring] *sites to support linear patient flows* [… with] *separate entrances and exit* *'*;^28^ however, vaccination programmes are considerably different to undifferentiated presentations to GPs.

### Implications for research and practice

The action taken by practices in the South West can inform future decisions about how practices can reduce infection risk for patients and staff, while maintaining responsibility for their own patients, rather than directing patients to specific locations (hot hubs) for treatment.

Eliciting patient views about when face-to-face contact is necessary, whether measures taken by practices provided reassurance that their buildings were safe environments, and whether the minimisation of face-to-face contact was acceptable, are critical to striking the correct balance between risk and need in the future.

How practices reconfigured their services depended on individual differences in their estates and facilities that are not typically captured systematically. Future research could explore how practice characteristics, such as list size and deprivation, relate to differences between practice estates and facilities and their ability to cope with the pandemic.

One key finding was the level of innovation and creative flexibility used by general practices to respond very rapidly to the new and challenging circumstances of the pandemic. The general practice response was aided by daily guidance provided by the CCG and One Care (the local GP federation) working closely together. This highlights the importance of balancing centralised guidance with allowing local flexibility in implementation and recognises the ability of relatively small organisations, like general practices, to respond rapidly and creatively during a crisis. Furthermore, it is an important lesson to carry over as practices continue to adjust their services as they recover from the pandemic. Future research could explore the forms of help and guidance that were helpful or unhelpful in supporting general practices during the pandemic.

COVID-19 is likely to change the way general practice works for up to 2 years and future pandemic situations are likely.^29^ The very different ways that practices in BNSSG organised face-to-face care to minimise infection highlights learning for the future for low-prevalence pandemic regions, who wish to keep all services in-house to maintain continuity of care.
